# Complete mitochondrial genomes of four entomopathogenic nematode species of the genus *Steinernema*

**DOI:** 10.1186/s13071-016-1730-z

**Published:** 2016-08-05

**Authors:** Taisei Kikuchi, Tanzila Afrin, Mutsuhiro Yoshida

**Affiliations:** 1 Division of Parasitology, Faculty of Medicine, University of Miyazaki, Miyazaki, 889-1692 Japan; 2 Kyushu Okinawa Agricultural Research Center, National Agriculture and Food Research Organization, Kumamoto, 861-1192 Japan

**Keywords:** Mitochondria, Genome, Insect parasitic nematode

## Abstract

**Background:**

Nematodes belonging to the genus *Steinernema* are insect parasites and are used as effective biological agents against soil-dwelling insect pests. Although the full nuclear genomes of multiple *Steinernema* species have become available recently, mitochondrial genome information for the genus is limited. In this study, we sequenced the complete mitochondrial genomes of four species of *Steinernema* and analysed their structure, codon usage and phylogenetic relationships.

**Results:**

Mitochondrial genomes of *Steinernema carpocapsae*, *S. glaseri*, *S. kushidai* and *S. litorale* comprised 13,924, 13,851, 15,182 and 21,403 bp, respectively, with highly AT-rich nucleotide contents (AT ratio of 71.05–76.76 %). All the expected genes, including 12 protein-coding genes (encoding ATP6, CYTB, COX1-3, ND1-6 and ND4L), two rRNA genes and 22 tRNA genes were identified in the four genomes. Phylogenetic analyses based on the amino acid sequences of the 12 protein-coding genes identified the *Steinernema* species as monophyletic, representing a sister clade of Rhabditina and Ascaridida. In addition, they were more closely positioned to other Clade 10 nematodes, including *Bursaphelenchus xylophilus*, *Aphelenchoides besseyi* and *Panagrellus redivivus*, than to *Strongyloides* species. Gene arrangements and codon usage analyses supported this relationship. Mitochondrial genome comparison of two distinct strains of *S. carpocapsae* detected high intra-specific diversity.

**Conclusions:**

The mitochondrial genomes of four species of *Steinernema* determined in this study revealed inter- and intra-species divergences/diversities of mitochondrial genomes in this genus. This information provides useful insights into the phylogenetic position of the genus *Steinernema* within the Nematoda and represents a useful resource for selecting molecular markers for diagnosis and population studies. These data will increase our understanding of the interesting biology of insect parasites.

**Electronic supplementary material:**

The online version of this article (doi:10.1186/s13071-016-1730-z) contains supplementary material, which is available to authorized users.

## Background

Nematodes belonging to the genus *Steinernema* are insect parasites that are used as biological agents to control soil-dwelling insect pests [1, 2]. *Steinernema* nematodes form symbiotic associations with enterobacteria of the genus *Xenorhabdus* and the ability to kill insects is formed by a complex association of the nematodes and the bacteria, which makes the nematodes and the bacteria an attractive model for studying animal-microbe symbiosis [3, 4].

*Steinernema* nematodes belong to infraorder Panagrolaimomorpha, which includes, among others, the vertebrate-parasitic genus *Strongyloides* and free-living bacteriovores of the genera *Panagrolaimus* and *Panagrellus* [5]. Phylogenetic reconstruction based on the small subunit ribosomal RNA gene placed the *Steinernema* spp. in Clade IV [6] and, in a later study, in Clade 10 [7] with the Panagrolaimomorpha nematodes and the plant-parasitic/fungivorous order Aphelenchida, which includes *Bursaphelenchus xylophilus* and *Aphelenchoides besseyi*.

The number of species in the genus has increased rapidly in recent years because of extensive surveys around the world that aimed to identify more efficient biological control agents (for examples, see [8–10]). Currently, about 80 described species can be found in the genus in the NCBI taxonomy database (http://www.ncbi.nlm.nih.gov/Taxonomy/). Considering the great diversity of hosts (insects), further specific diversity in this nematode group is expected.

Mitochondrial genome sequences have been widely used in population or evolutionary studies of nematodes. Mitochondrial genomes provide a rich source of molecular variation and widespread utility in population genetics and evolutionary biology [11]. Furthermore, mitochondria are deeply involved in many biological processes, from energy production to programmed cell death and ageing [12]. Studying mitochondrial genomes is, therefore, essential for our understanding of fundamental biology of eukaryotes.

Recently, the full nuclear genomes of five *Steinernema* species were sequenced [13], which paved the way to investigate the population structures and genetic factors involved in their key biological processes and parasitism using genome-wide analyses. By contrast, mitochondrial genome information was limited to only one species, *S. carpocapsae* [14]. Complete mitochondrial genome sequences from other *Steinernema* species will certainly be an important resource to study their ecology and parasitic biology. In this study, we sequenced the complete mitochondrial genomes of four species of *Steinernema* and analysed their structure, codon usage and phylogenetic relationships.

## Methods

### Biological materials

*Steinernema carpocapsae* (strain All), *Steinernema glaseri* (strain Sds102), *Steinernema kushidai* (strain Hamakita) and *Steinernema litorale* (strain IbKt142) were maintained using insect larvae (*Galleria mellonella* or *Anomala cuprea*) at the laboratory of plant nematology of Meiji University or Kyushu Okinawa Agricultural Research Center, National Agriculture and Food Research Organization. Infective third-stage juveniles of the nematodes were isolated from the infected insect cadavers using a White trap and further purified by the Berman funnel method, as described previously [15, 16].

### Library preparation and sequencing

Genomic DNA was extracted from the nematodes using a QIAamp DNA Mini Kit (Qiagen, Tokyo, Japan) according to the manufacturer’s instructions. Illumina libraries were constructed using a Nextera DNA Sample Prep Kit (Illumina, San Diego, USA) with 100 ng of DNA, according to the manufacturer’s instructions. The libraries were sequenced on an Illumina MiSeq using a v3 Reagent kit (600 cycles), according to the manufacturer’s recommended protocol (https://icom.illumina.com/) to produce 300-bp paired-end reads.

### Mitochondrial genome assembly

Mitochondrial genomes were reconstructed from the Illumina reads using MITObim ver. 1.6 [17]. Initial assemblies were generated using SGA assembler [18] and mitochondrial fragments in the assembly were identified by BlastX using *Caenorhabditis elegans* mitochondrial genes as queries. Those fragments were extended by iterative mapping of Illumina short reads using MITObim [14]. Gap regions in the assemblies were PCR amplified using Tks Gflex DNA Polymerase (Takara, Shiga, Japan) and sequenced on an ABI 3130 sequencer with a BigDye Terminator v3.1 Cycle Sequencing Kit (Applied Biosystems, Foster City, USA) or Illumina MiSeq, as mentioned above, to obtain a complete mitochondrial genome sequence. The assembled mitochondrial genomes were annotated for protein-coding, tRNA and rRNA genes using the MITOS web server [19] and by manual curation, with support from sequence similarity to other published mitochondrial genomes using Artemis genome annotation tool [20]. Repeats in the assemblies were detected using Tandem Repeat Finder ver.4.09 [21]. General statistical values were calculated using R (ver3.1.1) and in-house Python scripts.

### Ribosomal RNA sequencing

Full-length 18S ribosomal RNA genes were amplified using primers 988F (5′-CTC AAA GAT TAA GCC ATG C-3′) and 2646R (5′-GCT ACC TTG TTA CGA CTT TT-3′) [7]. The PCR products were purified using an innuPREP PCRpure kit (Analytik Jena, Jena, Germany) and sequenced on the ABI 3130 sequencer with the BigDye Terminator v3.1 Cycle Sequencing Kit (Applied Biosystems).

### Phylogenetic analysis

Thirty-nine nematode mitochondrial genomes were selected and included in the analysis, with an emphasis on species from Holterman’s Clade 10, to which *Steinernema* nematodes belong. Amino acid alignments for 12 protein-coding genes were generated separately using MAFFT [22], with options (−L-INS-i), and trimmed by Gblocks [23] with less stringent options to remove non-well-aligned sites. The best substitution model for each alignment was estimated using ProtTest [24]. All the protein alignments were concatenated and maximum likelihood trees were constructed using RaxML ver. 7.2.8 [25], under the MtArt model, for each gene partition. The tree topologies obtained from ML analyses were evaluated with 1,000 bootstrap pseudoreplications. Bayesian inference was performed using MrBayes 3.2.2 [26] from 0.5 million Markov Chain Monte Carlo generations, under a strict clock model using the Mtrev model for each gene partition. Markov chains were sampled at intervals of 100 generations. The first 0.1 million generations were discarded as ‘burn-in’ and three independent Markov Chain Monte Carlo runs converged to the same posterior probability.

Sequences of the small subunit ribosomal RNA gene were aligned using MAFFT version 7 [22], with option E-INS-I, and a maximum likelihood tree was obtained using RaxML ver. 7.2.8 [25] under the GTR-gamma model with 500 bootstrap pseudoreplications.

## Results

### Mitochondrial genome assembly

**Fig. 1:**
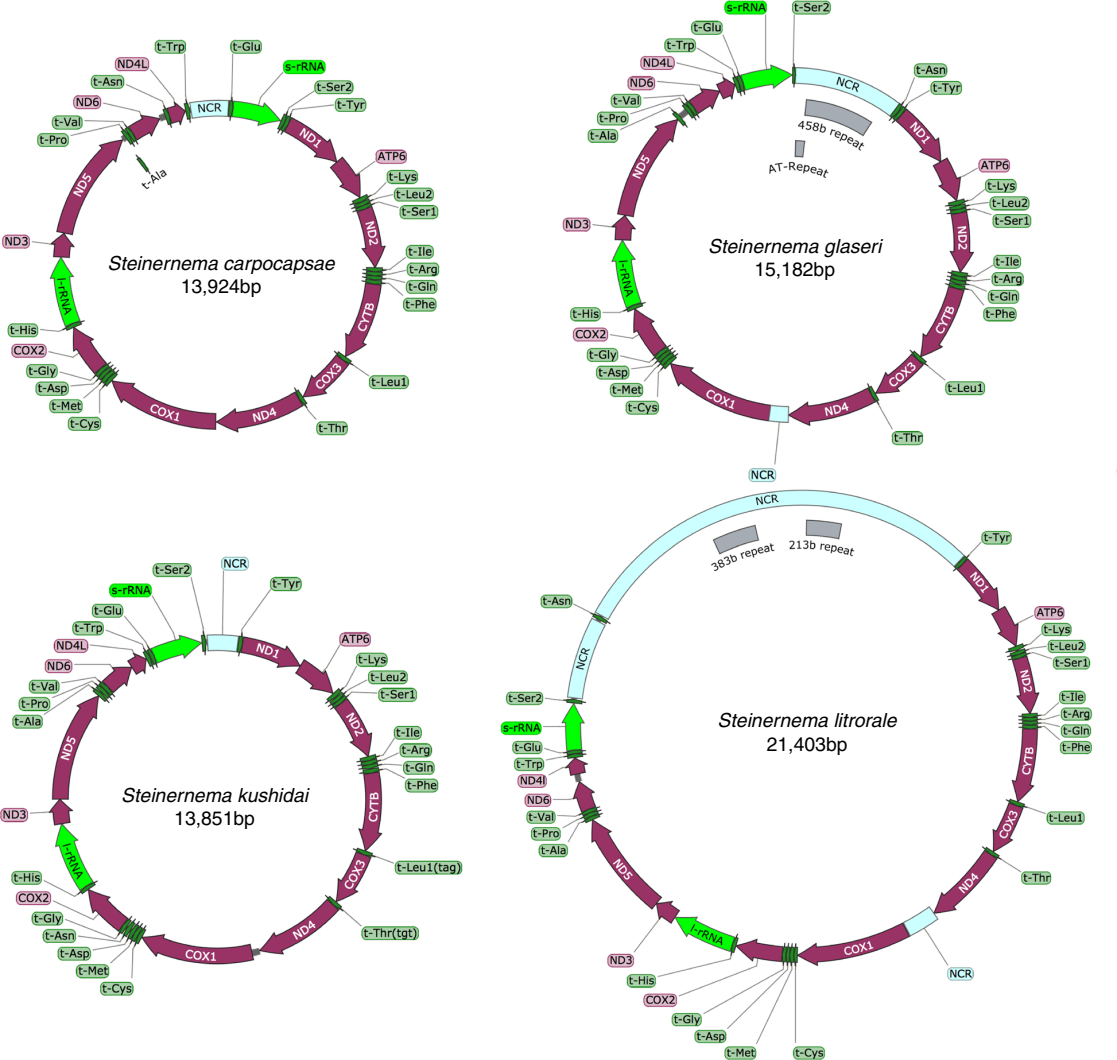
Schematic overview of the four mitochondrial genomes

We sequenced mitochondrial genomes of four *Steinernema* species: *S. carpocapsae*, *S. glaseri*, *S. kushidai* and *S. litorale*. The assembly resulted in single circular molecules for all four species with genome sizes ranging from 14 to 21 kb (13,924, 13,851, 15,182 and 21,403 bp for *S. carpocapsae*, *S. kushidai*, *S. glaseri* and *S. litorale*, respectively) (Table 1). This size difference mainly reflected the length of the non-coding regions in the genomes. *Steinernema litorale* and *S. glaseri* had large non-coding regions (8,137 and 1,962 bp, respectively), while the non-coding regions of *S. carpocapsae* and *S. kushidai* were shorter than 1 kb (Fig. 1, Table 1). Similar to mitochondrial genomes of other species, the nucleotide compositions were highly AT rich (AT ratio of 71.05–76.76 %). *Steinernema kushidai* and *S. litorale* had a slightly lower AT % than the other two species. Non-coding regions showed higher AT %s than coding regions (Table 1). All four mitochondrial genomes contain 12 protein-coding genes (encoding ATP6, CYTB, COX1-3, ND1-6 and ND4L), two rRNA genes and 22 tRNA genes (Fig. 1). Like most other nematodes, an *atp8* gene was not found in the genomes. All 36 genes were encoded on the same strand.

**Table 1:** General statistics for four *Steinernema* mitochondrial genomes

	*S. carpocapsae*	*S. glaseri*	*S. kushidai*	*S. litorale*
Length (bp)	13,924	15,182	13,851	21,403
Number of protein coding genes	12	12	12	12
Length of protein coding genes (bp)	10,309	10,336	10,315	10,395
Number of tRNAs	22	22	22	22
tRNA length (bp)	1,243	1,233	1,249	1,234
Number of rRNAs	2	2	2	2
rRNA length (bp)	1,648	1,651	1,644	1,637
Non-coding region length (bp)	724	1,962	643	8,137
AT% total	76.36	76.76	71.91	71.05
AT% protein coding region	76.22	75.71	70.86	69.79
AT% tRNA	74.09	74.53	71.9	72.53
AT% rRNA	76.94	75.89	75.43	71.9
AT% non-coding region	80.93	84.43	79.77	72.26

Comparison of the protein-coding genes between the *Steinernema* spp. showed that the *atp6*, *cox1* and *cox3* genes (mean pairwise distances 17.64, 16.09 and 17.73 %, respectively) were more conserved than *nad2* (27.45 %), *nad3* (24.57 %) and *nad5* (24.02 %) in those species (Table 2).

**Table 2:** Divergence of encoded proteins in the four *Steinernema* species. Pairwise p-distances were calculated using the Distmat program at the amino acid level

	Mean distance %)	Maximum distance (%)	Minimum distance (%)
ATP6	17.64	20.07	15.17
CYTB	20.08	22.31	17.43
COX1	16.09	17.86	14.44
COX2	18.40	21.79	15.37
COX3	17.73	19.53	15.36
ND1	19.00	20.62	18.1
ND2	27.45	29.98	25.36
ND3	24.57	27.83	21.13
ND4	23.13	26.84	13.85
ND5	22.10	24.65	19.72
ND6	24.02	26.7	21.34
ND7	22.88	25.69	20.92

### Codon usage

Codon usages of the four mitochondrial genomes are shown in Additional file 1: Table S1. In *S. glaseri*, *S. litorale* and *S. kushidai*, ATT was the most frequently used start codon (seven, five and eight cases, respectively) followed by TTG, ATG and ATA. Two GTTs in *S. kushidai* were the only start codons that were exceptions to the translation table (transl_table 5; invertebrate mitochondrial code). By contrast, in *S. carpocapsae*, exceptional start codons were used frequently (TTT for six genes and TTA for two genes). ATT and ATA start codons were observed in only two genes each in *S. carpocapsae*. TAA and TAG were used as termination codons in many of the genes. A truncated stop codon T was also observed in a small number of genes in all four species. The most frequently used codon in all four species was TTT (phe) followed by ATT (Ile) and TTA (leu). Principal component analysis (PCA) suggested that the overall codon usage patterns were similar in the four species compared with other Clade10 nematodes (Additional file 2: Figure S1). Within the *Steinernema*, the pattern of *S. carpocapsae* was similar to that of *S. glaseri*, whereas *S. litorale* showed a similar pattern to *S. kushidai* (Additional file 2: Figure S1).

### Non-coding regions

The *S. glaseri* mitochondrial genome had a long non-coding region (1,525 bp). The non-coding region was highly AT rich (AT ratio of 85.71 %) and contained repeat sequences. Tandem repeat finders identified a short tandem repeat (112 copies of “AT”) and a long repeat (two copies of the 458-bp unit) in the sequence (Fig. 1, Additional file 1: Table S2). Two long non-coding sequences were found in the *S. litorale* mitochondrial genome: one between tRNA-Ser2 and tRNA-Asn (1,249 bp), and the other between tRNA-Asn and tRNA-Tyr (6,260 bp) (Fig. 1). The latter region contained many repeat sequences with variable unit sizes, with the longest unit being 383 bp (Additional file 1: Table S2).

### Phylogenetic analysis

**Fig. 2:**
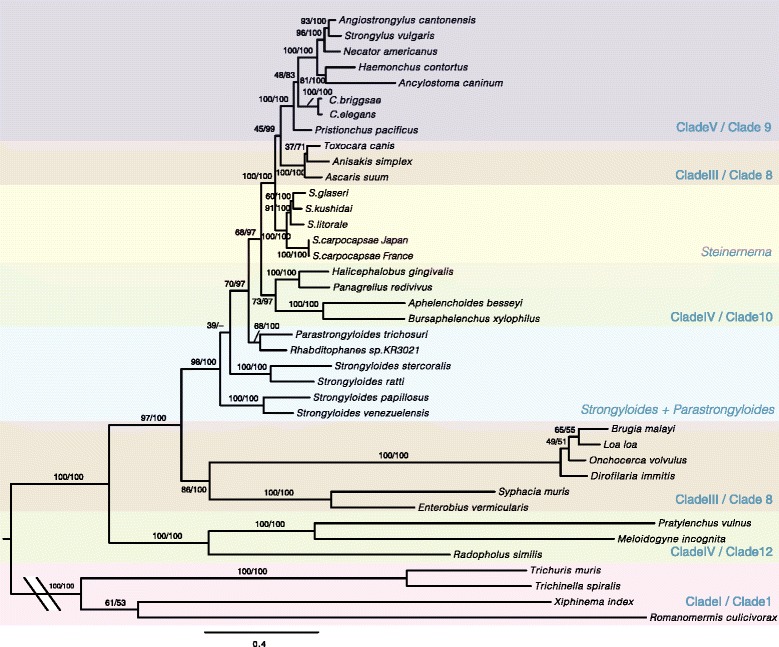
Maximum-likelihood tree inferred from 12 mitochondrial proteins. Amino acid sequences were aligned before concatenation, and the phylogenetic analysis was performed using RAxML v7.2.8 with 500 bootstrap resampling replicates. Almost the same topology was obtained from the same amino acid alignment using Bayesian analysis with MrBayes v3.2.2. Numbers above the branches indicate bootstrap values for maximum-likelihood and Bayesian posterior probabilities, respectively. The scale-bar shows the number of amino acid substitutions per site

A maximum likelihood tree based on 12 protein-coding genes from 39 nematode mitochondrial genomes, with enoplean nematode sequences as outgroups, is shown in Fig. 2. Bayesian analysis showed identical topologies, except for the relationship within *Strongyloides* species: in the Bayesian tree, *S. ratti* and *S. stercoralis* occupied more basal positions than *S. venezuelensis* and *S. papillosus*. The four *Steinernema* species formed a monophylic group as a sister clade of the Rhabditina (Holterman’s Clade 9) and the Ascaridida (Holterman’s Clade 8) species, with high support. Clade 10 species, including *B. xylophilus*, *A. besseyi*, *P. redivivus* and *Halicephalobus gingivalis*, together, were placed at the base of the *Steinernema* plus Rhabditina and Ascaridida clade. *Strongyloides* species were placed at a position further outside of them.

Within the *Steinernema* clade, *S. carpocapsae* occupied the basal position and *S. glaseri* plus *S. kushidai* was at the most derived position. The relationships of the four *Steinernema* species inferred from the nuclear 18S rRNA gene were slightly different (Additional file 2: Figure S2). In the 18S rRNA gene tree, *S. litorale* and *S. kushidai* were positioned most internally, although *S. carpocapsae* was placed at the base of the four species.

### Gene arrangements

**Fig. 3:**
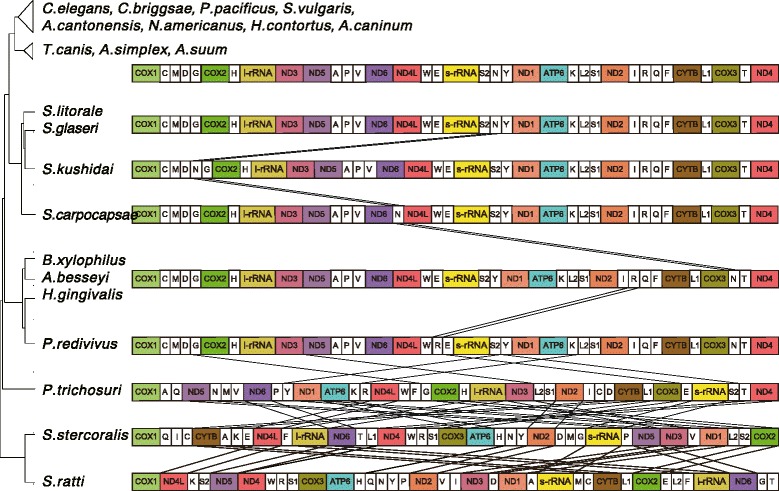
Mitochondrial gene arrangements in *Steinernema* nematodes and other related species. Genes and non-coding regions are not scaled. All 36 genes are transcribed from left to right. Lines between the horizontal bars represent possible rearrangement events

All the genes in the *Steinernema* mitochondrial genomes were transcribed in one direction, as in other Chromadorea nematodes (Fig. 3). The gene arrangements were also well conserved within the *Steinernema* species and Rhabditina species, including *C. elegans* and *Pristionchus pacificus*, and Ascaridida species, including *A. suum* and *Toxocara canis. Steinernema glaseri* and *S. litorale* showed perfectly identical gene arrangements to *C. elegans*, whereas *S. kushidai* and *S. carpocapsae* were different only in the position of tRNA-His (Fig. 3). Other Clade 10 nematodes, including *B. xylophilus*, *A. besseyi, P. redivivus* and *H. gingivalis*, showed similar gene arrangements to the *Steinernema* spp. with only few rearrangements of tRNA genes. *Strongyloides* clade species (including *Strongyloides* spp. and *Parastrongyloides trichosuri)* were the exception, showing highly rearranged mitochondrial genomes [27].

### Intraspecific variation

A mitochondrial genome of a different strain of *S. carpocapsae* was previously reported (strain Breton, originating from France) [14]. Comparison of the French sequence with our *S. carpocapsae* (strain All, originating from Japan) revealed intraspecific diversity. A nucleotide alignment identified 201 single nucleotide variants (SNV) and nine indel positions, which accounted to 1.5 % of the total genome. Among the 201 SNVs, 177 were located in protein-coding regions, 17 in the tRNA region, four in the rRNA region and three in non-coding regions (Table 3). Within the protein-coding regions, 146 variants were synonymous and 31 were non-synonymous. Indels were identified only in non-coding regions (six indels) and in the rRNA region (three indels) (Table 3).

**Table 3:** Distributions of single nucleotide variations and indels in *S. carpocapsae* mitochondrial gene

	SNVs	Indels
Protein-coding	177	0
Synonymous	146	–
Non-synonymous	31	–
tRNA	17	0
rRNA	4	3
Non-coding region	3	6
Total	201	9

## Discussion

Although the full nuclear genomes of five *Steinernema* species were published recently [13], information on the mitochondrial genomes of this genus was limited. In this study, we sequenced four complete mitochondrial genomes using next-generation sequencing techniques. To the best of our knowledge, this is the first study employing multi-species comparison of mitochondrial genomes in this genus.

Inconsistencies between mitochondrial and nuclear rRNA gene phylogenies of Nematoda have been reported previously [27, 28]. The mitochondrial gene tree obtained in this study showed a similar topology to those in previous studies. For example, the non-monophyly of Clade III (*sensu* Blaxter et al. 1998 [6]) and the non-monophyly of Tylenchomorpha (*sensu* De Ley & Blaxter, 2002 [5]) were also observed in our tree. In our mitochondrial gene phylogeny, the four *Steinernema* species were clustered as a sister clade of Rhabditina plus Ascaridida, with strong support (Fig. 2). Although *Steinernema* and *Strongyloides* have been placed in the same superfamily (Strongyloidoidea), the phylogeny did not support closer relationships between the two genera. This finding agrees with recent studies [14, 28–30]. In this study, we included more *Steinernema* species and Clade 10 species in the phylogenetic analysis, which confirmed the distant relationship of *Steinernema* spp. to *Strongyloides* spp.

Comparisons of codon usage or gene arrangements are useful to obtain insights into phylogenetic relationships. The codon usage in *Steinernema* species was distinguishable from that of other related species in the PCA plot (Additional file 2: Figure S1). The gene arrangements suggested that *Steinernema* is more closely related to Rhabditina and Ascaridida species than to *Strongyloides* species (Fig. 3). These results are consistent with the phylogeny based on amino acid data. However, it is not straightforward to interpret the relationships of the four species in the genus. The codon usage analysis placed *S. glaseri* and *S. carpocapsae* in a sub-group (Additional file 2: Figure S1); however, the gene arrangements of *S. glaseri* were more similar to *S. litorale* than to *S. carpocapsae* (Fig. 3). Either of these results are not completely consistent with mitochondrial amino acid phylogeny and 18S phylogeny (Fig. 2; Additional file 2: Figure S2).

Comparison of two strains of *S. carpocapsae* of different origins revealed 1.5 % variations in the mitochondrial genomes at the nucleotide level, indicating that the mitochondrial genome can provide good markers for population studies. We observed a location-biased distribution of SNVs and indels, suggesting that marker selection will be important for population studies. Interestingly, the rRNA gene regions have fewer SNVs than the protein-coding region, while indels were more frequently represented in the rRNA gene and were not found in protein-coding regions. This is probably because of functional differences between protein-coding and rRNAs. Indels in protein-coding region can be more problematic because they could cause large protein changes by insertions of stop or frame-shift mutations, whereas mismatches by SNVs, rather than indels, could be critical in rRNA genes because they could produce more erratic RNA secondary structures. These are only the results from a comparison of a small number of strains. For a deep understanding of their evolution patterns, it will be interesting to re-sequence and compare more mitochondrial genomes from a larger numbers of strains using high-throughput sequencers. The genome information provided in this study will be useful as references in those kinds of studies.

## Conclusions

In this study, the complete mitochondrial genome sequences of four *Steinernema* species were determined. The complete mitochondrial genomes of *S. carpocapsae*, *S. glaseri*, *S. kushidai* and *S. litorale* were 13,924, 13,851, 15,182 and 21,403 bp in length, respectively, and all contained the 36 expected genes (12 protein-coding genes, two ribosomal RNA genes and 22 transfer RNA genes), all transcribed in the same direction. Phylogenetic analyses using the amino acid sequences of the 12 protein-coding genes showed that the *Steinernema* species formed a monophylic clade as a sister clade of Rhabditina and Asacridida. They were also more closely positioned to other Clade 10 nematodes, including *B. xylophilus*, *A. besseyi* and *P. redivivus*, than to *Strongyloides* species, which belong to the same infraorder as the *Steinernema* species. The data on codon usage and gene arrangement also supported this result. We also compared the mitochondrial genomes of two strains of *S. carpocapsae* and detected high intraspecific diversity. This mitochondrial genome information will form a useful resource for evolutionary and population studies of *Steinernema* nematodes.

## Abbreviations

Not applicable.

## Additional files

Additional file 1: Table S1. Codon usage frequencies in the four *Steinernema* mitochondrial genomes. Table S2. Tandem repeats in large non-coding regions (> 1 kb) in *S. glaseri* and *S. litorale*. (PDF 78 kb) Additional file 2: Figure S1. Principal component analysis of the codon usage in *Steinernema* mitochondrial genes. The first (PC1) and the second principal component (PC2) account for 98.1 % of the variability. *Abbreviations*: S.car, *S. carpocapsae*; Sgla, *S. glaseri*; Skus, *S. kushidai*; Slit, *S. litorale*; Bxyl, *B. xylophilus*; Pred, *P. redivivus*; Ptri, *P. trichosuri*. Figure S2. Phylogenetic relationships among *Steinernema* species inferred from nearly full-length nuclear 18S rRNA genes. A maximum-likelihood tree based on nucleotide sequences, with *Panagrellus redivivus* as the outgroup, was generated with the substitution model TVM+ G. Bootstrap values are given on the branches. The scale-bar represents the number of nucleotide substitutions per site. (PDF 28 kb)

## References

[1] Burnell AM, Stock SP (2000). *Heterorhabditis*, *Steinernema* and their bacterial symbionts lethal pathogens of insects. Nematology.

[2] Lacey LA, Georgis R (2012). Entomopathogenic nematodes for control of insect pests above and below ground with comments on commercial production. J Nematol.

[3] Murfin KE, Dillman AR, Foster JM, Bulgheresi S, Slatko BE, Sternberg PW (2012). Nematode-bacterium symbioses - Cooperation and conflict revealed in the ‘omics’ age. Biol Bull.

[4] Martens EC, Heungens K, Goodrich-Blair H (2003). Early colonization events in the mutualistic association between *Steinernema carpocapsae* nematodes and *Xenorhabdus nematophila* bacteria. J Bacteriol.

[5] De Ley P, Blaxter M, Lee DL (2002). Systematic Position and Phylogeny. The Biology of Nematodes.

[6] Blaxter ML, De Ley P, Garey JR, Liu LX, Scheldeman P, Vierstraete A (1998). A molecular evolutionary framework for the phylum Nematoda. Nature.

[7] Holterman M, van der Wurff A, van den Elsen S, van Megen H, Bongers T, Holovachov O (2006). Phylum-wide analysis of SSU rDNA reveals deep phylogenetic relationships among nematodes and accelerated evolution toward crown Clades. Mol Biol Evol.

[8] San-Blas E, Portillo E, Nermut J, Puza V, Morales-Montero P (2015). *Steinernema papillatum* n. sp. (Rhabditida: Steinernematidae), a new entomopathogenic nematode from Venezuela. Nematology.

[9] Ma J, Chen S, De Clercq P, Waeyenberge L, Han R, Moens M (2012). A new entomopathogenic nematode, *Steinernema xinbinense* n. sp. (Nematoda: Steinernematidae), from north China. Nematology.

[10] Cimen H, Lee M-M, Hatting J, Hazir S, Stock SP (2014). *Steinernema tophus* sp. n. (Nematoda: Steinernematidae), a new entomopathogenic nematode from South Africa. Zootaxa.

[11] Galtier N, Nabholz B, GlÉMin S, Hurst GDD (2009). Mitochondrial DNA as a marker of molecular diversity: a reappraisal. Mol Ecol.

[12] Balaban RS, Nemoto S, Finkel T (2005). Mitochondria, oxidants, and aging. Cell.

[13] Dillman AR, Macchietto M, Porter CF, Rogers A, Williams B, Antoshechkin I (2015). Comparative genomics of *Steinernema* reveals deeply conserved gene regulatory networks. Genome Biol.

[14] Montiel R, Lucena MA, Medeiros J, Simoes N (2006). The complete mitochondrial genome of the entomopathogenic nematode *Steinernema carpocapsae*: insights into nematode mitochondrial DNA evolution and phylogeny. J Mol Evol.

[15] White GF (1927). A method for obtaining infective nematode larvae from cultures. Science.

[16] Ogura N, Nakashima T (1997). Cold tolerance and preconditioning of infective juveniles of *Steinernema kushidai* (Nematoda: Steinernematidae). Nematologica.

[17] Hahn C, Bachmann L, Chevreux B (2013). Reconstructing mitochondrial genomes directly from genomic next-generation sequencing reads-baiting and iterative mapping approach. Nucleic Acids Res.

[18] Simpson JT, Durbin R (2012). Efficient de novo assembly of large genomes using compressed data structures. Genome Res.

[19] Bernt M, Donath A, Juhling F, Externbrink F, Florentz C, Fritzsch G (2013). MITOS: Improved de novo metazoan mitochondrial genome annotation. Mol Phylogenet Evol.

[20] Carver T, Harris SR, Berriman M, Parkhill J, McQuillan JA (2012). Artemis: an integrated platform for visualization and analysis of high-throughput sequence-based experimental data. Bioinformatics.

[21] Benson G (1999). Tandem repeats finder: a program to analyze DNA sequences. Nucleic Acids Res.

[22] Katoh K, Misawa K, Kuma K, Miyata T (2002). MAFFT: a novel method for rapid multiple sequence alignment based on fast Fourier transform. Nucleic Acids Res.

[23] Talavera G, Castresana J (2007). Improvement of phylogenies after removing divergent and ambiguously aligned blocks from protein sequence alignments. Syst Biol.

[24] Abascal F, Zardoya R, Posada D (2005). ProtTest: selection of best-fit models of protein evolution. Bioinformatics.

[25] Stamatakis A (2006). RAxML-VI-HPC: maximum likelihood-based phylogenetic analyses with thousands of taxa and mixed models. Bioinformatics.

[26] Huelsenbeck JP, Ronquist F (2001). MRBAYES: Bayesian inference of phylogenetic trees. Bioinformatics.

[27] Hunt VL, Tsai IJ, Coghlan A, Reid AJ, Holroyd N, Foth BJ (2016). The genomic basis of parasitism in the *Strongyloides* clade of nematodes. Nat Genet.

[28] Park J-K, Sultana T, Lee S-H, Kang S, Kim HK, Min G-S, Eom KS, Nadler SA (2011). Monophyly of clade III nematodes is not supported by phylogenetic analysis of complete mitochondrial genome sequences. BMC Genomics.

[29] Kim T, Kim J, Nadler SA, Park J-K (2016). The complete mitochondrial genome of *Koerneria sudhausi* (Diplogasteromorpha: Nematoda) supports monophyly of Diplogasteromorpha within Rhabditomorpha. Curr Genet.

[30] Kim J, Lee S, Gazi M, Kim T, Jung D, Chun J, Kim S, Seo T, Park C, Baldwin JG (2015). Mitochondrial genomes advance phylogenetic hypotheses for Tylenchina (Nematoda: Chromadorea). Zool Scripta.

