# Supplementary material for: Complete mitochondrial genomes of four entomopathogenic nematode species of the genus Steinernema
Source: Parasit Vectors. 2016 Aug 5;9:430. doi: 10.1186/s13071-016-1730-z (PMC4974692; doi:10.1186/s13071-016-1730-z)
Supplement: Additional file 1: Table S1. — Codon usage frequencies in the four Steinernema mitochondrial genomes. Table S2. Tandem repeats in large non-coding regions (> 1 kb) in S. glaseri and S. litorale. (PDF 78 kb) [file 13071_2016_1730_MOESM1_ESM.pdf]

Table S1. Codon usage frequencies in the four *Steinernema* mitochondrial genomes.

AminoAcid	Codon	*Steinernema carpocapsae*	*Steinernema glaseri*	*Steinernema kushidai*	*Steinernema litorale*
A	GCA	2 (0.02)	10 (0.09)	3 (0.03)	4 (0.04)
A	GCC	16 (0.17)	12 (0.11)	19 (0.18)	12 (0.11)
A	GCG	3 (0.03)	4 (0.04)	9 (0.09)	4 (0.04)
A	GCT	71 (0.77)	82 (0.76)	74 (0.7)	92 (0.82)
C	TGC	1 (0.02)	3 (0.06)	9 (0.2)	6 (0.13)
C	TGT	51 (0.98)	47 (0.94)	37 (0.8)	39 (0.87)
D	GAC	8 (0.13)	3 (0.05)	9 (0.14)	20 (0.31)
D	GAT	56 (0.88)	62 (0.95)	54 (0.86)	45 (0.69)
E	GAA	54 (0.68)	54 (0.7)	34 (0.45)	22 (0.3)
E	GAG	25 (0.32)	23 (0.3)	41 (0.55)	52 (0.7)
F	TTC	24 (0.04)	36 (0.07)	66 (0.14)	54 (0.12)
F	TTT	561 (0.96)	457 (0.93)	420 (0.86)	406 (0.88)
G	GGA	8 (0.04)	26 (0.13)	16 (0.08)	15 (0.08)
G	GGC	4 (0.02)	2 (0.01)	9 (0.05)	12 (0.06)
G	GGG	19 (0.10)	13 (0.07)	28 (0.15)	35 (0.18)
G	GGT	151 (0.83)	158 (0.79)	136 (0.72)	130 (0.68)
H	CAC	5 (0.09)	2 (0.03)	14 (0.24)	6 (0.11)
H	CAT	51 (0.91)	57 (0.97)	44 (0.76)	51 (0.89)
I	ATC	7 (0.02)	15 (0.05)	48 (0.17)	40 (0.17)
I	ATT	281 (0.98)	281 (0.95)	237 (0.83)	200 (0.83)
K	AAA	64 (0.76)	62 (0.66)	59 (0.64)	49 (0.53)
K	AAG	20 (0.24)	32 (0.34)	33 (0.36)	43 (0.47)
L	CTA	21 (0.04)	15 (0.03)	35 (0.07)	33 (0.06)
L	CTC	10 (0.02)	7 (0.01)	22 (0.04)	15 (0.03)
L	CTG	5 (0.01)	5 (0.01)	31 (0.06)	40 (0.08)
L	CTT	104 (0.22)	114 (0.22)	162 (0.32)	128 (0.24)
L	TTA	255 (0.53)	288 (0.55)	163 (0.32)	194 (0.37)
L	TTG	82 (0.17)	92 (0.18)	92 (0.18)	115 (0.22)
M	ATA	121 (0.78)	132 (0.77)	131 (0.72)	133 (0.75)
M	ATG	35 (0.22)	39 (0.23)	51 (0.28)	44 (0.25)
N	AAC	14 (0.1)	10 (0.07)	31 (0.23)	35 (0.27)
N	AAT	125 (0.9)	138 (0.93)	106 (0.77)	94 (0.73)
P	CCA	10 (0.12)	18 (0.21)	18 (0.21)	10 (0.12)
P	CCC	6 (0.07)	8 (0.09)	5 (0.06)	8 (0.09)
P	CCG	2 (0.02)	2 (0.02)	8 (0.09)	10 (0.12)
P	CCT	65 (0.78)	57 (0.67)	54 (0.64)	58 (0.67)
Q	CAA	35 (0.83)	31 (0.76)	16 (0.37)	14 (0.33)
Q	CAG	7 (0.17)	10 (0.24)	27 (0.63)	28 (0.67)
R	CGA	1 (0.03)	1 (0.03)	2 (0.06)	0 (0.00)
R	CGC	1 (0.03)	0 (0.00)	0 (0.00)	0 (0.00)
R	CGG	0 (0.0)	1 (0.03)	1 (0.03)	0 (0.00)
R	CGT	30 (0.94)	29 (0.94)	28 (0.9)	31 (1.0)
S	AGA	54 (0.14)	63 (0.17)	51 (0.12)	49 (0.12)
S	AGC	7 (0.02)	4 (0.01)	21 (0.05)	27 (0.06)
S	AGG	18 (0.05)	19 (0.05)	46 (0.11)	44 (0.11)
S	AGT	133 (0.34)	127 (0.34)	114 (0.28)	121 (0.29)
S	TCA	20 (0.05)	22 (0.06)	21 (0.05)	16 (0.04)
S	TCC	7 (0.02)	9 (0.02)	10 (0.02)	6 (0.01)
S	TCG	1 (0.00)	7 (0.02)	7 (0.02)	13 (0.03)
S	TCT	150 (0.38)	126 (0.33)	143 (0.35)	140 (0.34)
T	ACA	23 (0.20)	25 (0.22)	20 (0.17)	29 (0.22)
T	ACC	6 (0.05)	9 (0.08)	9 (0.08)	8 (0.06)
T	ACG	1 (0.01)	2 (0.02)	10 (0.08)	11 (0.08)
T	ACT	83 (0.73)	77 (0.68)	79 (0.67)	85 (0.64)
V	GTA	38 (0.15)	46 (0.19)	39 (0.15)	67 (0.22)
V	GTC	13 (0.05)	4 (0.02)	25 (0.10)	28 (0.09)
V	GTG	12 (0.05)	17 (0.07)	21 (0.08)	52 (0.17)
V	GTT	193 (0.75)	179 (0.73)	177 (0.68)	163 (0.53)
W	TGA	41 (0.58)	55 (0.76)	35 (0.49)	37 (0.51)
W	TGG	30 (0.42)	17 (0.24)	36 (0.51)	35 (0.49)
Y	TAC	18 (0.1)	12 (0.07)	38 (0.22)	57 (0.31)
Y	TAT	154 (0.9)	164 (0.93)	131 (0.78)	128 (0.69)
start	ATA	2 (0.17)	2 (0.17)	0 (0.00)	1 (0.08)
start	ATC	0 (0.00)	0 (0.00)	1 (0.08)	0 (0.00)
start	ATG	0 (0.00)	1 (0.08)	1 (0.08)	2 (0.17)
start	ATT	2 (0.17)	7 (0.58)	5 (0.42)	8 (0.67)
start	GTT	0 (0.00)	0 (0.00)	2 (0.17)	0 (0.00)
start	TTA	2 (0.17)	0 (0.00)	0 (0.00)	0 (0.00)
start	TTG	0 (0.00)	2 (0.17)	3 (0.25)	1 (0.08)
start	TTT	6 (0.5)	0 (0.00)	0 (0.00)	0 (0.00)
stop	T	1 (0.08)	1 (0.08)	1 (0.08)	3 (0.25)
stop	TAA	8 (0.67)	9 (0.75)	7 (0.58)	4 (0.33)
stop	TAG	3 (0.25)	2 (0.17)	4 (0.33)	5 (0.42)

Values in parentheses represent ratio for each amino acid.

Table S2. Tandem repeats in large non-coding regions (>1kb) in *S. glaseri* and *S. litorale*

S. glaseri

position in NCR1	Period size	Copy number	Consensus size	Percent matches	Percent indels	Score	A	C	G	T	Entropy (0-2)
33--242	2	112	2	83	14	288	48	0	0	50	1.04
190--1343	458	2.5	457	96	2	2096	42	6	9	41	1.62

S. litrale

position in NCR1	Period size	Copy number	Consensus size	Percent matches	Percent indels	Score	A	C	G	T	Entropy (0-2)
2137--2868	383	1.9	383	97	0	1383	35	8	13	42	1.73
3627--4303	2	373.5	2	67	25	307	48	1	2	46	1.26
3627--4303	11	62.3	12	68	23	307	48	1	2	46	1.26
3627--4100	208	2.3	208	79	14	516	48	1	2	47	1.23
3725--4308	231	2.5	233	87	9	806	48	1	3	46	1.28
4484--4542	12	5.4	11	75	19	52	49	1	0	49	1.11
4475--4542	10	6.8	10	76	21	63	50	1	0	48	1.1
4475--4545	22	3.3	22	72	22	69	50	1	0	47	1.09
4531--4667	33	4.2	33	73	11	122	40	9	5	43	1.61
