# Supplementary material for: Complete mitochondrial genomes of four entomopathogenic nematode species of the genus Steinernema
Source: Parasit Vectors. 2016 Aug 5;9:430. doi: 10.1186/s13071-016-1730-z (PMC4974692; doi:10.1186/s13071-016-1730-z)
Supplement: Additional file 2: Figure S1. — Principal component analysis of the codon usage in Steinernema mitochondrial genes. The first (PC1) and the second principal component (PC2) account for 98.1 % of the variability. Abbreviations: S.car, S. carpocapsae; Sgla, S. glaseri; Skus, S. kushidai; Slit, S. litorale; Bxyl, B. xylophilus; Pred, P. redivivus; Ptri, P. trichosuri. Figure S2. Phylogenetic relationships among Steinernema species inferred from nearly full-length nuclear 18S rRNA genes. A maximum-likelihood tree based on nucleotide sequences, with Panagrellus redivivus as the outgroup, was generated with the substitution model TVM+ G. Bootstrap values are given on the branches. The scale-bar represents the number of nucleotide substitutions per site. (PDF 28 kb) [file 13071_2016_1730_MOESM2_ESM.pdf]

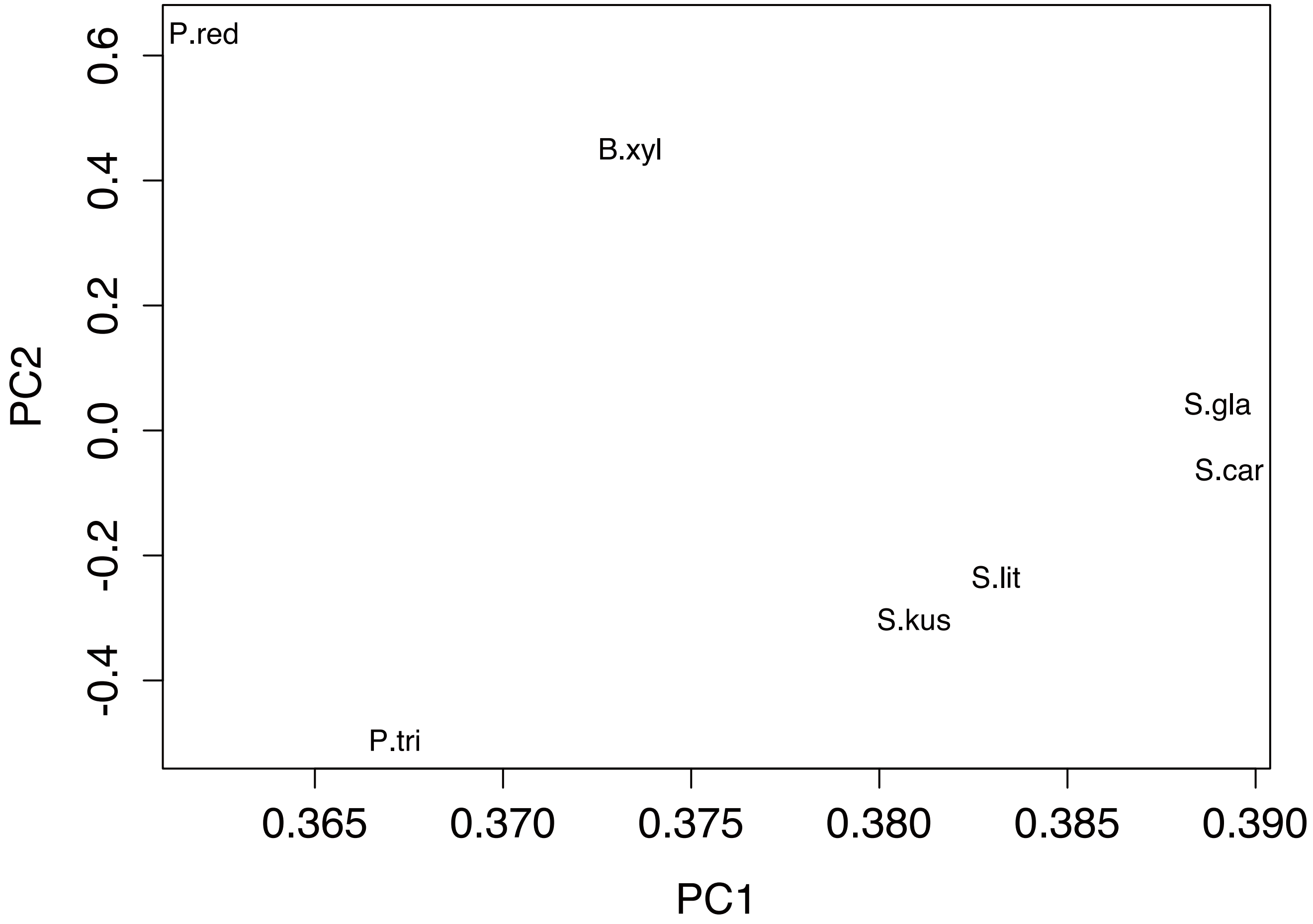

Fig. S1. Principal component analysis of the codon usage in Steinernema mitochondrial genes. The first PC (PC1) plus the second PC (PC2) account for 98.1% of the variability. S.car: S. carpocapsae, Sgla: S. glaseri, Skus: S. kushidai, Slit: S. litorale, Bxyl: B. xylophilus, Pred: P. redivivus, Ptri: P. trichosuri.

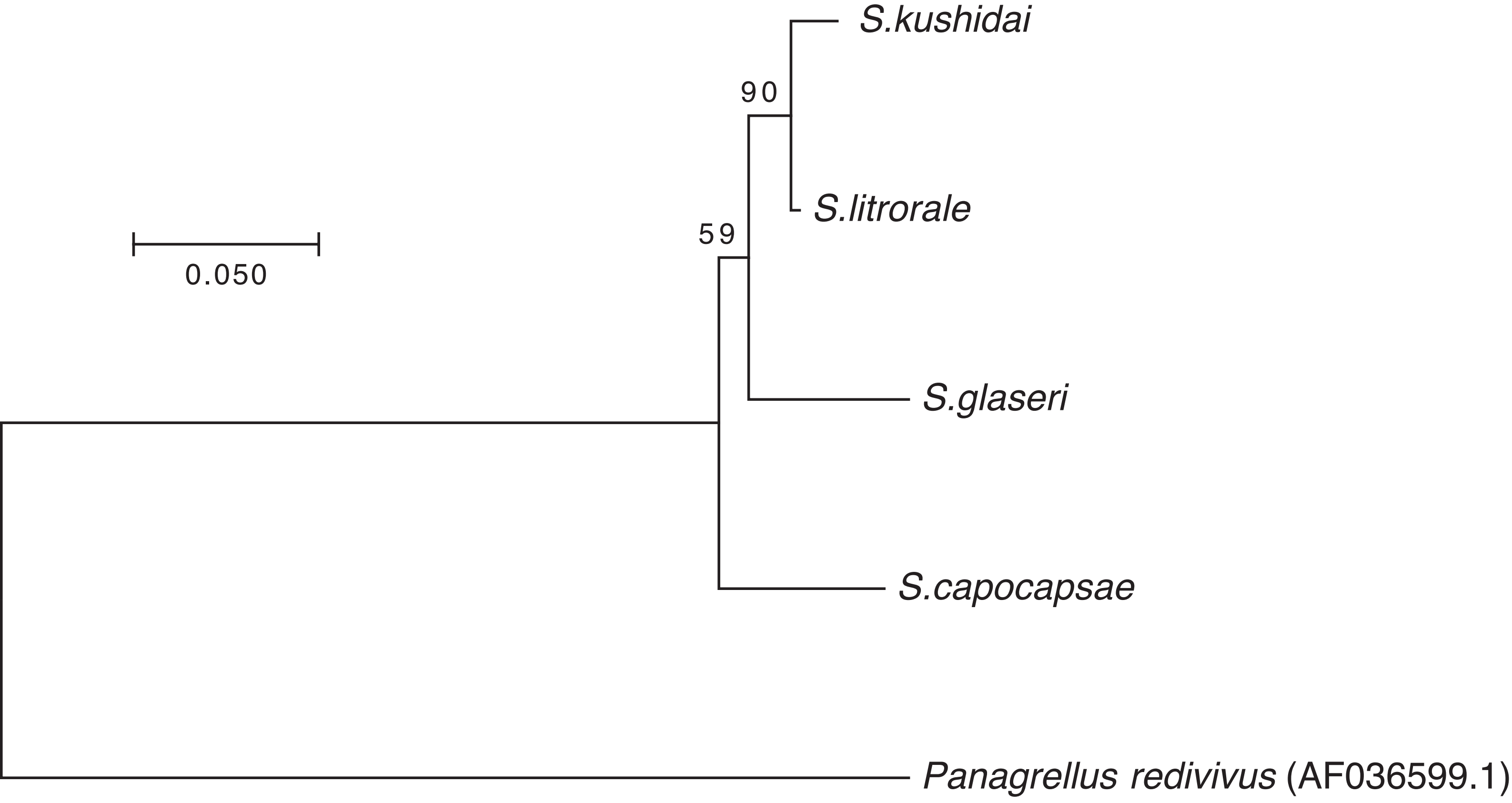

Fig. S2. Phylogenetic relationships among Steinernema species inferred from nearly full-length 18S rRNA genes.
A maximum-likelihood tree based on nucleotide sequences, with Panagrellus redivivus as an outgroup, was generated with the substitution model TVM+ G. Bootstrap values are given on the branches. The scale bar represents the number of nucleotide substitutions per site.
